# Alleviation of cognitive deficits in a rat model of glutamate-induced excitotoxicity, using an N-type voltage-gated calcium channel ligand, extracted from *Agelena labyrinthica* crude venom

**DOI:** 10.3389/fnmol.2023.1123343

**Published:** 2023-02-17

**Authors:** Mohammad Keimasi, Kowsar Salehifard, Mohammadjavad Keimasi, Mohammadreza Amirsadri, Noushin Mirshah Jafar Esfahani, Majid Moradmand, Fariba Esmaeili, Mohammad Reza Mofid

**Affiliations:** ^1^Department of Plant and Animal Biology, Faculty of Biological Sciences and Technology, University of Isfahan, Isfahan, Iran; ^2^Department of Physiology, School of Medicine, Isfahan University of Medical Sciences, Isfahan, Iran; ^3^Department of Clinical Pharmacy and Pharmacy Practice, School of Pharmacy and Pharmaceutical Sciences, Isfahan University of Medical Sciences, Isfahan, Iran; ^4^Department of Clinical Biochemistry, School of Pharmacy and Pharmaceutical Sciences, Isfahan University of Medical Sciences, Isfahan, Iran

**Keywords:** cognitive dysfunction, spider, calcium channel Cav2.2 (N-type), learning and memory, venom

## Abstract

Excitotoxicity is a common pathological process in Alzheimer’s disease (AD) which is caused by the over-activity of N-Methyl-D-Aspartate receptors (NMDARs). The release of neurotransmitters depends on the activity of voltage-gated calcium channels (VGCCs). Hyper-stimulation of NMDARs can enhance the releasement of neurotransmitters through the VGCCs. This malfunction of channels can be blocked by selective and potent N-type VGCCs ligand. Under excitotoxicity condition, glutamate has negative effects on the pyramidal cells of the hippocampus, which ends in synaptic loss and elimination of these cells. These events leads to learning and memory elimination through the hippocampus circuit’s dysfunction. A suitable ligand has a high affinity to receptor or channel and is selective for its target. The bioactive small proteins of venom have these characteristics. Therefore, peptides and small proteins of animal venom are precious sources for pharmacological applications. The omega-agatoxin-Aa2a was purified, and identified from *Agelena labyrinthica* specimens, as an N-type VGCCs ligand for this study. The effect of the omega-agatoxin-Aa2a on the glutamate-induced excitotoxicity in rats was evaluated through behavioral tests including Morris Water Maze, and Passive avoidance. The syntaxin1A (SY1A), synaptotagmin1 (SYT1), and synaptophysin (SYN) genes expression were measured *via* Real-Time PCR. The local expression of synaptosomal-associated protein, 25 k Da (SNAP-25) was visualized using an immunofluorescence assay for synaptic quantification. Electrophysiological amplitude of field excitatory postsynaptic potentials (fEPSPs) in the input–output and LTP curves of mossy fiber were recorded. The cresyl violet staining of hippocampus sections was performed for the groups. Our results demonstrated that the omega-agatoxin-Aa2a treatment could recover the learning, and memory impairment caused by NMDA-induced excitotoxicity in rat hippocampus.

## Introduction

Neurodegeneration is a symptom of central nervous system diseases like as Alzheimer’s disease (AD), Parkinson’s disease (PD), Huntington’s disease (HD), and Amyotrophic lateral sclerosis (ALS). Various circumstances such as aging, trauma or compression of brain tissue, extreme stress, oxidative stress, neuro-inflammation, and excitotoxicity can induce neurodegeneration (Przedborski et al., 2003; Mehta et al., 2013). During neurodegeneration, the neurons lose their functions over time, which leads to neuronal death through apoptosis and necrosis. This elimination can generate particular defects, based on the involved neurodegeneration region. These defects have a wide range and intensity; depend on level of the disease progression. Neurodegeneration in hippocampus area can cause amnesia, forgetfulness, and cognitive impairment, which ends in AD (Crews and Masliah, 2010). AD has the highest prevalence among the neurodegenerative disorders (Naseri et al., 2022; Sarlaki et al., 2022).

Alzheimer’s disease (AD) extensively increases direct (including both formal medical and non-medical care) and indirect (reduced productivity) health-related costs due to its effects on both quality of life and productivity of patients themselves as well as their caregivers. Also it is projected that AD costs increases due to the growth in its prevalence and treatment costs. The societal financial burden of AD is estimated to rise by about 4.9-fold in the USA (to $1.5 trillion), 2.7-fold in Europe (to €633 billion), and 9.5-fold worldwide (to $9.1 trillion), by 2050 (Dauphinot et al., 2022; Tahami Monfared et al., 2022). Consequently, finding new effective ways to prevent or treat AD can be very worthwhile when increasing health costs is taken into account.

Excitotoxicity is an important precondition for the neurodegeneration, and can be induced by the hyper-activation of N-Methyl-D-Aspartate receptors (NMDARs). This action has a variety of consequences on pyramidal neurons of the hippocampus such as production of high amounts of various free radicals, mitochondrial dysfunction, and activation of apoptotic factors and caspases, which leads to neuro-termination (Rahn et al., 2012). Higher than normal rates of neurotransmitters in synapses are essential for hyper-activation of NMDARs (Arundine and Tymianski, 2003). The major excitatory neurotransmitter in hippocampus is glutamate, which has a crucial role in memory, and learning (Bin Ibrahim et al., 2022). Synaptic firing through the hippocampus trisynaptic circuit is essential for memory formation (Alkadhi, 2021). Also, long-term potentiation (LTP) is a vital process for memory performance (Hayashi, 2022). These processes rely on the release of neurotransmitters. Release of the neurotransmitters is the most important role of voltage-gated calcium channels (VGCCs) (Nimmrich and Gross, 2012). N-type VGCCs are located in the presynaptic terminal and are activated by an action potential. Following activation, calcium ions enter through the N-type VGCCs, which, in turn leads to neurotransmitter release (Sousa et al., 2013). Synaptosomal-associated protein, 25 k Da (SNAP-25) belongs to the soluble N-ethylmaleimide-sensitive factor activating protein receptor protein superfamily, and contributes to exocytosis. SNAP-25 plays a key role in intracellular vesicle trafficking which is crucial for neurotransmitter release (Zhang et al., 2014; Biswal et al., 2017; Li and Kavalali, 2017). Therefore, the SNAP-25 can be used as a synaptic marker. Synaptotagmin 1 (SYT1) is a synaptic calcium sensor, which regulate neurotransmitter release. This sensor has an undeniable role in vesicle exocytosis, and neurotransmitters release in synaptic cleft (Zhang et al., 2014; Hussain et al., 2017). The rate of SYT1 decreases in AD (Li and Kavalali, 2017). Synaptophysin (SYN) is a synaptic marker with a fundamental role in synapse functions including vesicle trafficking, and neurotransmitters release (Zhang et al., 2014; Ji et al., 2017). The syntaxin1A (SY1A) is localized in the presynaptic membrane and with other synaptic proteins triggers the docking of synaptic vesicles (Kim and Oh, 2016). The SY1A has a direct link with SNAP-25 (Ullrich et al., 2015). High rates of glutamate in synapses caused excitotoxicity (Dong et al., 2009). Therefore, the normal activation of N-type VGCCs is necessary for the prevention of excitotoxicity consequences. Use of suitable ligands can reveal the impact of N-type VGCCs in excitotoxicity process. The venom and its components are valuable as some approaches to the mentioned goal (Sousa et al., 2013).

Venom of animals such as snails, snakes, scorpions, and spiders are valuable sources of peptides, and small proteins with unique specifications, such as high affinity to their target receptors or channels, great selectivity, stable structure, and low molecular mass (Lewis and Garcia, 2003; Moradi et al., 2018). Therefore, these ligands can be used for the correction of malfunction of over-activated channels. In this case, the omega-agatoxin-Aa2a as an N-type VGCCs ligand can demonstrate the impact of N-type VGCCs in neurodegenerative disorders, particularly AD. This ligand can be found in Agelenidae family. *Agelena labyrinthica* species is a member of Agelenidae family (Herzig et al., 2010; Consortium, 2019).

We could not find any direct and comprehensive study regarding the effects of N-type VGCCs on the excitotoxicity of glutamate, as a cognitive impairment *in vivo* model. The aim of this study was to evaluate the effects of neurotransmitters release on memory, and learning impairments. To achieve this goal, behavioral, molecular, electrophysiological, and histological methods were used in the current study. The *Agelena labyrinthica* spiders were collected from Iran. The epigyne, fangs, and venom glands were observed with a stereoscope. Then the collected venom of these spiders were extracted from venom glands and lyophilized. After that, gel-electrophoresis, gel-filtration chromatography, and capillary electrophoresis were performed for purification and subsequently, mass spectrometry (HPLC-ESI-MS) was used for identification of this ligand. Behavioral tasks including the Morris water maze, and passive avoidance were performed to assess learning, and memory. The SY1A, SYT1, and SYN genes expression were measured *via* Real-time PCR. In addition, the amount of SNAP-25 localized protein for synaptic quantification was measured through the immunofluorescence technique in the CA_3_ sub-region of the hippocampus. Then, the field excitatory postsynaptic potentials (fEPSP) amplitude after LTP in the Mossy Fiber pathway were recorded for evaluation of spatial memory, and memory formation. At last, the coronal section of hippocampus and particularly CA_3_ sub-region were stained through cresyl violet and visualized for comparison of pyramidal neuron in the experimental groups.

## Materials and methods

### Chemicals, reagents

All chemicals were purchased from Sigma Aldrich Company (Darmstadt, Germany) except for the others mentioned in the text.

### Spider collection, identification, and venom extraction

For this study, specimens were collected alive from Iran (Zamani et al., 2021). The *Agelena labyrinthica* was photographed with a camera. The specimens were kept under suitable conditions, humidity (60%), and temperature (25°C) and fed by crickets and mealworms. The specimens were identified using the taxonomic keys (Nentwig et al., 2017). Epigyne of the female specimen was visualized with a stereoscope.

Female spiders were separated to extract the venom. Specimens were anesthetized with CO_2_ in a small chamber, and the opisthosoma and carapace were removed under the stereoscope. The fangs and venom glands of the female specimen were visualized by a loop. Venom glands were collected into 4°C Phosphate buffered saline (PBS) were prepared in the laboratory with this recipe (137 mM NaCl, 3 mM KCl, 10 mM Na_2_PO_4_, 2 mM KH_2_PO_4_, and pH 7.4) and gently crushed with a glass stirrer for 30 min. Then, pieces of the venom gland were removed from the solution by centrifugation at 13,000 rpm for 30 min at 4°C, and the supernatant was lyophilized and stored at −70°C. Protein concentration was measured by Bradford assay with bovine serum albumin as standard protein.

### Determination of lethal dose (LD_50_)

To determine the LD_50_, the albino mice (average weight 18–20 g) were intravenously (IV) injected with crude venom. After the injection, the animals were followed up for 1 day. The Spearman-Karber method was used to calculate the LD_50_ dose for the crude venom and omega-agatoxin-Aa2a protein (Hamilton et al., 1977).

### SDS-PAGE of the crude venom

1 mg of the crude venom was dissolved in 1 ml PBS buffer and incubated at 4°C overnight. The supernatant was then centrifuged at 13,000 rpm for 4 min at 4°C. Then, 15 μl of dissolved crude venom was mixed with 5 μl loading buffer (1x) and heated for 10 min at 100°C. An amount of 20 μl of this solution was loaded into each well of the gel [15% Sodium Dodecyl Sulfate-Polyacrylamide Gel-Electrophoresis (SDS-PAGE)] which stained with coomassie blue dye for 45 min at 25°C. Hot water and a shaker were used to decolorize the gel. Finally, the gels were scanned by Bio-5000 Gel scanner device (Seyfi et al., 2019).

### Protein purification with gel-filtration chromatography

The lyophilized crude venom (10 mg) was resuspended in 1.5 mL of PBS buffer. The DNase (0.14 mg/ml) and RNase (0.14 mg/ml) enzymes were added to the sample and incubated for 2 h at 4°C. The clear solution was injected into a gel-filtration column (GE Healthcare HiLoad 16/600 Superdex^®^ 75 pg prep grade) and run over it using FPLC (Fast Protein Liquid Chromatography) system (Sykam, Germany). The column was washed with PBS Buffer. The injection volume was 1,200 μL and the flow rate was 0.7 mL/min. The fractions were observed with absorbance at 280 nm and collected in a 0.75 ml fraction. The chosen fractions (marked on the graph) were collected and injected into capillary electrophoresis and gel-electrophoresis (Mofid et al., 2021).

### Protein purification and separation with capillary electrophoresis:

Capillary electrophoresis test was performed by Agilent 7100 equipped with a UV-Vis detector using a 50 μm uncoated silica column with a total length of 40 cm and a detector distance of 8.5 cm from the outlet. PBS buffer with pH 4.7 was used for both the sample, and running buffer. The capillary temperature was 25°C and the sample was injected at 100 mBar for 5 s. Electrophoresis was performed for 5 min at 25 kV normal polarity. The marked peaks were collected, and protein concentration was determined by Bradford assay. The desired peak was re-injected into the device to ensure purity, and the result was presented the collected fraction was subjected to 12% SDS-PAGE. The selected peak obtained from this method project into HPLC-ESI-MS (Keimasi et al., 2022).

### Protein identification with mass spectrometry (HPLC-ESI-MS)

The high-performance liquid chromatography/electrospray ionization tandem mass spectrometry (HPLC-ESI-MS) analysis was performed by Waters Alliance 2695 HPLC-Micromass Quattro micro API Mass Spectrometer. Liquid chromatography separation was performed on Atlantis T3-C18 column (3 μ, 2.1 × 100 mm) at 35°C. Mobile phases were 0.1% formic acid in acetonitrile (A) and 0.1% formic acid in H_2_O. The gradient profile was 5% A held for 0.2 min and linearly increased to 90% in 10 min. Then, it was held for 5 min, which decreased to 5% over 3 min and finally held for 4 min. The flow rate was 0.2 mL/min, and the injection volume was 5 μL. The mass spectrometry method was included in positive mode with the capillary voltage, which was adjusted to 0.3 kV, and the source and dissolving temperatures were set at 120°C and 300°C respectively, with flow gas 200 L/h. The result was presented for the purified bio-active small protein peak (Jafari et al., 2014).

### Animals and experimental design

54 adult male Wistar rats, weighing 230–250 g were taken from the animal’s nest of the Faculty of Biological Sciences and Technology, University of Isfahan. They were kept in standard cages with controlled temperature (∼25°C) and humidity (∼40%), 12 h light; 12 h dark cycle, and free access to enough food and water. The ethics committee of the University of Isfahan approved the study.

The work was performed on male Wistar rats, which were spat into three groups (18 rats in each group). A small area on each rat’s skull was shaved while the head was fixed using a stereotaxic instrument (Stoelting Co., USA) to prepare for injection into the hippocampus. The PBS buffer was used as a vehicle for omega-agatoxin-Aa2a and N-Methyl d-Aspartate (NMDA). Rats were assigned into the following groups:

Control Group: Received 1 μl of PBS in the CA3 sub-region of the hippocampus twice and with an interval of 30 min.

NMDA-treated group: received a single dose of NMDA (1 μl, 10 μg/μl) in the CA3 sub-region of the hippocampus followed by 1 μl of PBS 30 min later (Jarrard, 2002).

Omega-agatoxin-Aa2a -treated group: received a single dose of NMDA (1 μl, 10 μg/μl) in the CA3 sub-region of the hippocampus followed by 1 μl of omega-agatoxin-Aa2a (2 μg/μl) 30 min later (Sousa et al., 2013).

### Surgery and microinjection procedure

One week before the start of the behavioral tests, all the animals were deeply anesthetized with an intraperitoneal (i.p.) injection of phenobarbital (40 mg/kg). The phenobarbital was purchased from Martindale Pharma Company (Buckinghamshire, England). The animals were wrapped in towels and the eyes were covered with Vaseline during the operation. Then, the CA_3_ sub-region of the hippocampus (AP:−3.3 mm from bregma; ML: ± 3 mm from midline; DV: 3.5 mm from the skull surface) was found using the Paxinos and Watson rat brain atlas (Paxinos and Watson, 2006). The injections were performed bilaterally on the hippocampi by stereotaxic apparatus.

The agents were bilaterally administered into the hippocampi using an injection needle (21 gauge) connected to a 5 μl Hamilton syringe through a polyethylene tube. The agents were slowly injected into the hippocampus area for 6 min. To avoid backflow of the fluid, the needle was slowly removed 2 min after the injection.

### Behavioral studies

The behavioral evaluations including the Morris water maze and Passive avoidance tasks were performed a week after the stereotaxic surgery. These tasks have assessed spatial, long-term memory, and learning. Before the behavioral tests, animals were acclimatized over 2 days in the laboratory area to be adjusted to the experimental conditions and to minimize stress.

### Morris water maze task

The Morris water maze test is one of the most common procedures to assess spatial memory and learning performance in rodents. The walls of the water maze room must have signs and symbols that rats can use their spatial memory to find the hidden platform in the target zone. Morris water maze task has three parts including habituation, training, and testing (it has been completely described in previous work) (Hosseini-Sharifabad et al., 2021). In this task, time spent in the target quadrant, distance moved in the target quadrant, the entry into the target quadrant, the velocity of rats, and swimming paths of rats in the last time of training were recorded to evaluate spatial memory and learning performance (ten rats in each group). All behavioral parameters of the rats were monitored by a video camera, fixed to the ceiling above the center of the pool and connected to a computerized tracking system (Auto vision Software, Designed by BorjSanat Company, Tehran, Iran).

### Passive avoidance task

The passive avoidance task was applied to assess non-conceptual communication memory and learning, as this method has already been used in neurological disorders models in small laboratory animals (Salehifard et al., 2023). The term passive avoidance is commonly used to describe experiments in which animals learn to avoid a painful stimulus. A passive avoidance behavior study was performed on two consecutive days (training and test), as explained by Ebrahimpour et al. (2018). The latency time before entering the dark section, and the time spent in the dark section on the test day, were recorded to evaluate passive avoidance learning and memory (10 rats in each group).

### RNA extraction, complementary DNA (cDNA) synthesis, and quantitative real-time PCR

The mRNA extraction was performed as formerly has been described by Gheysarzadeh et al. (2019) and mRNA expression measurement was done for the three groups (six rats in each group) (Gheysarzadeh et al., 2019). Total RNA was extracted from the hippocampus samples using RNX-PLUS reagent (SinaClon, Iran). cDNA was synthesized according to the manufacturer’s protocol with a cDNA synthesis kit (Takara, Japan). Real-time PCR assay was performed by StepOnePlus™ Real-Time PCR System. The sequences of Real-time PCR primers were as follows: 5′-GAGGAAGGTCTGAACCGCTCAT-3′, and 5′- CGTTCTCGGTAGTCTGACTGAG-3′ for mice syntaxin1A, 5′- AGGGCCTATGATGGACTTTCTG-3′, and 5′-TCCGTGGCCA TCTTCACATC-3′ for mice Synaptophysin, 5′-CGGCAAA CTGACTGTCATTC-3′ and 5′-GCC CCA GTG CTG TTG TAA CCA-3′ for mice Synaptotagmin 1, and 5′-CAGGG CTGCCTTCTCTTGTG-3′, and 5′-GATGGTGATGGGTTTC CCGT-3′ for mice Glyceraldehyde-3-phosphate dehydrogenase (GAPDH). Relative genomic expression was calculated by the 2^–Δ Δ Ct^ method. The mRNA levels were normalized to that of GAPDH (Gheysarzadeh et al., 2019).

### Electrophysiological study

The rats (six in each group) were anesthetized with urethane (1.5 g/kg; intraperitoneal injection) dissolved in 0.9% normal saline solution. The surgery and LTP recording procedures from the hippocampal CA_3_ area were executed as formerly has been described by Keimasi et al. (2022).

### Immunohistochemistry

The brains were washed with saline and sequentially fixed in 10% formaldehyde solution and then post-fixed in 15% and 25% sucrose solutions. Two-micrometer sections were cut by using a microtome instrument. Antigen retrieval was performed enzymatically for 20 min. To reduce non-specific antibody binding, the samples were blocked with the blocking agent [10% normal goat serum (Sigma, G9023) and 0.3% Triton X-100 in PBS] for 30 min at 37°C. After washing, tissue sections were incubated with mouse anti−SNAP25 monoclonal (Abcam, ab66066) as the primary antibody at 4°C. FITC−conjugated anti−mouse IgG (Sigma, F9137) were used as secondary antibody for 2 h at room temperature. The nuclei were counterstained with DAPI (Sigma, D9542). The slides were visualized using an AX70 Olympus fluorescence microscope (three in each group).

### Cresyl violet staining

Tissue coronal slides were deparaffinized two times in xylene, each for 10 min. Rehydration was performed using gradient alcohol (100, 95, 70, and 50% alcohol) each time for 5 min. The brain slides were stained in cresyl violet solution (0.25% cresyl violet, 0.8% glacial acetic acid, 0.6 mM sodium acetate) for 20 min. The dehydration was done using gradient alcohol (70, 95, and 100% alcohol) each time for 3 min. Tissue slides were visualized using a BX40 Olympus light microscopy (three in each group).

### Data analysis

Statistical analysis was performed using Graph Pad Prism statistics software (version 8.4.3). Statistical Data were evaluated by the D’Agostino-Pearson omnibus test to examine the normal distribution. The data collected were analyzed by one-way, and one-way repeated measures analysis of variance (ANOVA). For multiple comparisons, Tukey’s test was used. All data were shown as the mean ± standard errors of the means (*P* < 0.05 was considered a significant difference).

## Results

### Spider collection, identification, and venom extraction

**FIGURE 1:**
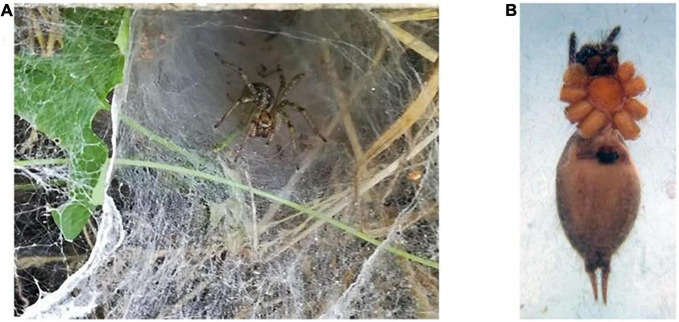
*Agelena labyrinthica* species. **(A)** The *Agelena labyrinthica* spider on the net. **(B)** The epigyne of female specimens.

A total of 150 spiders were collected from Iran (Zamani et al., 2021), and separated by species and gender. One hundred female *Agelena labyrinthica* specimens were selected for the next phase of the study. The specimens on the net web and epigyne of this species are shown in Figure 1.

**FIGURE 2:**
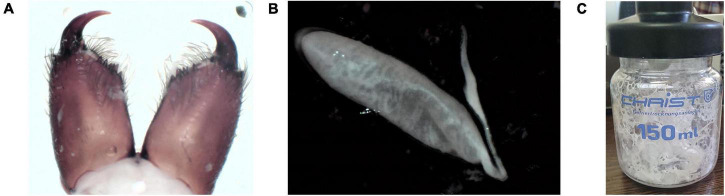
Venom extraction and venom glands of *Agelena labyrinthica*. **(A)** The *Agelena labyrinthica* fangs. **(B)** The venom gland. **(C)** The lyophilized extracted venom.

1.03 g of the lyophilized crude venom was extracted from *Agelena labyrinthica* specimens. Then, 10 mg of the crude venom was dissolved in PBS buffer. The protein concentration of the *Agelena labyrinthica* lyophilized crude venom was 7.2 mg/ml, which was determined by the Bradford method. The fangs, venom glands, and lyophilized crude venom of *Agelena labyrinthica* are presented in Figure 2.

### Determination of LD_50_

Based on the results of IV injections of the crude venom and omega-agatoxin-Aa2a protein into the albino mice, the LD_50_ was determined as 32.68 mg/kg and not toxic.

### SDS-PAGE

**FIGURE 3:**
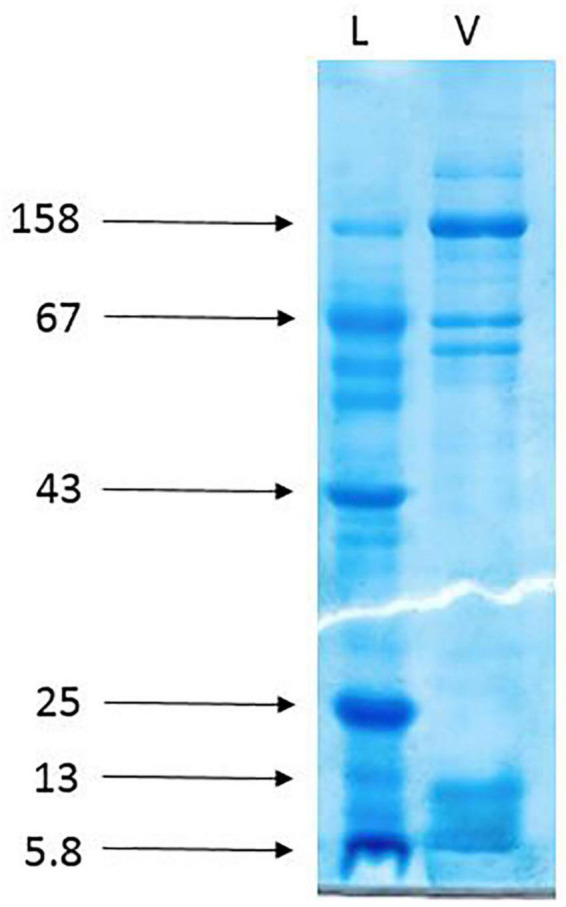
The gel electrophoresis of *Agelena labyrinthica* crude venom. L: Ladder (k Da), V: Venom.

The gel electrophoresis of the crude venom of *Agelena labyrinthica* showed a lot of protein bands separated based on their molecular weights. The strongest bands were the 158 and the band range of 3–10 k Da. The 3–10 k Da band was related to the agatoxin family, which has a molecular weight in this range (Herzig et al., 2010; Consortium, 2019; Figure 3).

### Protein purification with gel-filtration chromatography

**FIGURE 4:**
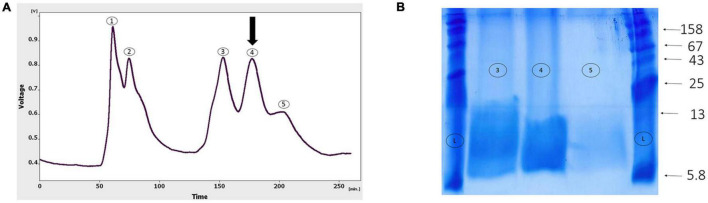
Gel-filtration chromatography of the *Agelena labyrinthica* venom, and the gel electrophoresis of the third, fourth, and fifth fractions. **(A)** Gel-filtration chromatography of the crude venom performed on a GE Healthcare HiLoad 16/600 Superdex^®^ 75 pg prep grade column in 1 M PBS (pH 7.4), flow rate 0.7 ml/min. The fourth fraction is shown by black arrow. **(B)** The gel electrophoresis of *Agelena labyrinthica*: 3, 4, and 5 fractions. L: Ladder (k Da).

As shown in Figure 4A, the gel-filtration chromatography of the *Agelena labyrinthica* venom had five peaks. The fourth fraction of *Agelena Labyrinthica* venom was collected from 160 to 190 min. 1.57 mg of the lyophilized fraction was obtained from 21 ml of the solution, taken from the instrument. The protein concentration of this fraction was 1.03 mg/ml.

The gel electrophoresis of the 4, 5, and 6 fractions are displayed in Figure 4B. This figure is completely consistent with the gel-filtration chromatography pattern, shown in Figure 4A.

### Protein purification with capillary electrophoresis (CE)

**FIGURE 5:**
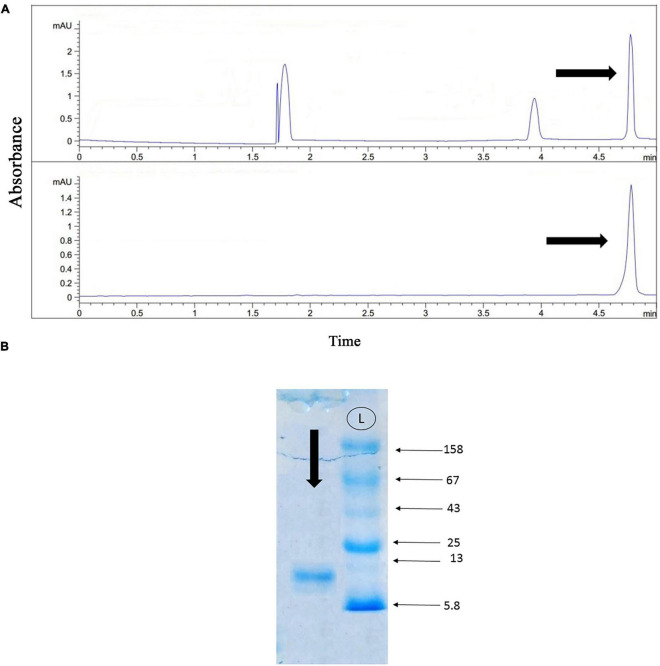
Capillary electrophoresis pattern of the fourth fraction, and the gel electrophoresis of the selected fraction. **(A)** Capillary electrophoresis of the fourth fraction was performed on a 50 μm uncoated silica column 1 M PBS (pH 4.7) for 5 min. The omega-agatoxin IIA fraction is shown by black arrow. **(B)** The gel electrophoresis of the selected peak. L: Ladder (k Da).

Figure 5, part A displays the capillary electrophoresis pattern, obtained from the fourth fraction of *Agelena labyrinthica* venom. Four peaks were observed at 1.71, 1.77, 3.95, and 4.78 min. The fourth peak was collected and re-injected to confirm the purity of this small bioactive protein and then injected into HPLC-ESI-MS for mass determination.

The gel electrophoresis of the selected peak revealed a single band of less than 13 kDa (Figure 5B).

### Protein identification with mass spectrometry (HPLC-ESI-MS)

**FIGURE 6:**
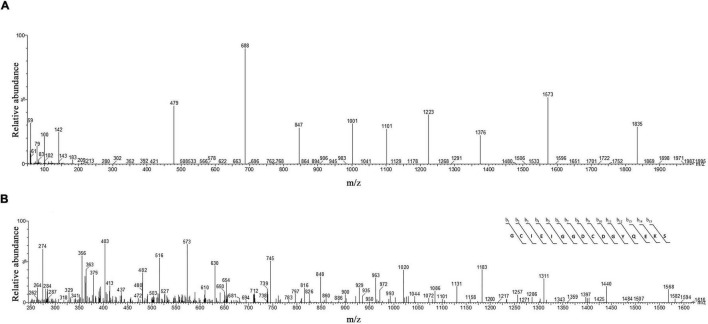
The identification of omega-agatoxin-Aa2a by mass spectrophotometry. **(A)** HPLC-ESI-MS of the selected peak from capillary electrophoresis was performed on Atlantis T3-C18 column. **(B)** N-terminal partial sequencing of the omega-agatoxin-Aa2a protein. The singly charged ion of the N-terminal peptide (m/z, 1406) was subjected to the fragmentation in the ion trap mass analyzer. The observed fragment ions are indicated above and below the peptide sequence.

The molecular mass of omega-agatoxin-Aa2a is 10,982 Da, according to the Sousa et al. (2013) study. The spectrum results demonstrated the existence of omega-agatoxin-Aa2a in the selected peak (Figure 6A). The mass-to-charge ratio of this ligand was consistent with omega-agatoxin-Aa2a. The charge-to-mass ratio of omega-agatoxin-Aa2a is as follows: (M + Na + 5H)^6+^ = 1835, (M + Na + 6H)^7+^ = 1573, (M + Na + 7H)^8+^ = 1,376.5. The Mass/Mass spectrum of Quadro charge ion at 1,376.5 was selected for N-terminal partial sequencing as follows: b_3_ = 274, b_4_ = 403, b_5_ = 516, b_6_ = 573, b_7_ = 630, b_8_ = 745, b_9_ = 848, b_10_ = 963, b_11_ = 1,020, b_12_ = 1,183, b_13_ = 1,311, b_14_ = 1,440, and b_15_ = 1,568. The results of the spectrum indicated a good consistency with the omega-agatoxin-Aa2a (Accession number: P15971) sequence (Figure 6B).

### Morris water maze task

**FIGURE 7:**
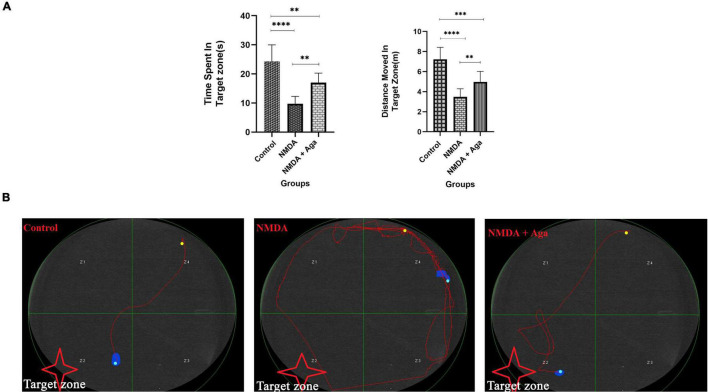
Morris water maze task. **(A)** The effect of treatment with omega-agatoxin-Aa2a on the time spent in the target zone. The data are shown as means ± SEM of 6 rats per group. (*P < 0.05, ^**^P < 0.01, ^***^P < 0.001, and ^****^P < 0.0001). **(B)** The swim path of the rats from the last time of training.

The intra-hippocampal administration of NMDA, significantly decreased the time spent in the target area (*P* < 0.0001), and distance moved in the target zone (*P* < 0.0001), when compared to the control group. NMDA treatment with administration of omega-agatoxin-Aa2a increased the time spent in the target zone (*P* < 0.01), and distance moved in the target zone (*P* < 0.01) of the maze, compared to the NMDA-treated group (*P* < 0.01). Also, significant differences in the time spent (*P* < 0.01), and distance moved (*P* < 0.01) in the target zone of the maze were observed between the control, and NMDA + Aga groups (Figure 7A).

The swimming path of experimental animals during the last time of training is displayed in Figure 7B. The swimming path of the control group was short and the rats found the hidden platform, easily. But, the swimming path of the NMDA group was long and the rats failed to locate the hidden platform. The majority of the movements of these rats were limited to the area close to the wall of the pool. However, the swimming path of the NMDA-treated with a single dose of omega-agatoxin-Aa2a group was almost short and the evaluated rats were able to find the hidden platform after a quick exploration in the water pool.

### Passive avoidance task

**FIGURE 8:**
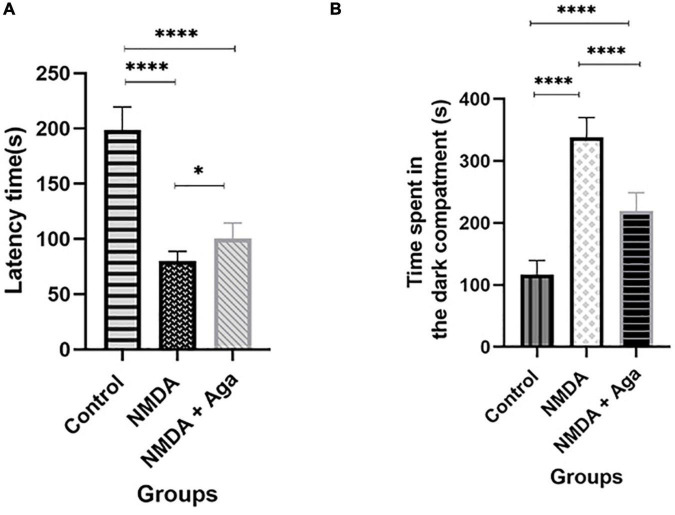
The passive avoidance task. **(A)** The effect of treatment with omega-agatoxin-Aa2a on the latency time before entering the dark section in the test day. The data are shown as means ± SEM of 6 rats per group. (*P < 0.05, ^**^P < 0.01, ^***^P < 0.001, and ^****^P < 0.0001). **(B)** The effect of treatment with omega-agatoxin-Aa2a on the time spent in the dark compartment in the test day. The data are shown as means ± SEM of 6 rats per group. (*P < 0.05, ^**^P < 0.01, ^***^P < 0.001, and ^****^P < 0.0001).

According to Figure 8A, our data revealed that administration of a single dose of NMDA into the hippocampus, significantly decreases the latency time in the NMDA group, compared to the control group (*P* < 0.0001), in the passive avoidance task. NMDA treatment with a single dose of omega-agatoxin-Aa2a increased the latency time in the NMDA + Aga group compared to the NMDA-treated group (*P* < 0.05). The Control group was significantly different from the NMDA + Aga group (*P* < 0.0001).

As shown in Figure 8B, our results demonstrated that the intra-hippocampal injection of NMDA significantly increases the time spent in the dark chamber in the NMDA-treated group when compared to the control group (*P* < 0.0001). Accordingly, NMDA treatment with omega-agatoxin-Aa2a decreases the time spent in the dark chamber in the NMDA + Aga group compared to the NMDA-treated group (*P* < 0.0001), in the passive avoidance task. There is a significant difference between the NMDA + Aga and the control groups (*P* < 0.0001).

### Quantitative real-time PCR

**FIGURE 9:**
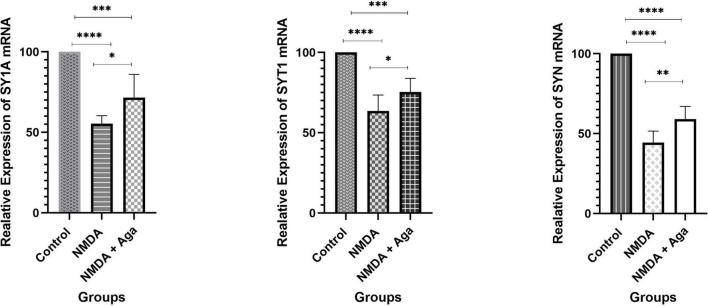
The quantitative real-time PCR. The effect of treatment with omega-agatoxin-Aa2a on the hippocampal SY1A, SYT1, and SYN mRNA levels. The data are shown as means ± SEM of six rats per group. (*P < 0.05 and ^**^P < 0.01 and ^***^P < 0.001 and ^****^P < 0.0001).

Figure 9 displays the effect of NMDA-treated and NMDA-treated + Aga groups on, SY1A, SYT1, and SYN genes expression in the rat hippocampus. This figure reveals that administration of NMDA in the rat hippocampus distinctly has decreased SY1A (*P* < 0.0001), SYT1 (*P* < 0.0001), and SYN (*P* < 0.0001) mRNAs expression when compared to the control group. The NMDA-treated group with a single dosage injection of omega-agatoxin-Aa2a increased SY1A (*P* < 0.05), SYT1 (*P* < 0.01), and SYN (*P* < 0.05) mRNAs expression with respect to the NMDA-treated group. Significant differences were observed between the two examined groups (mRNAs expression: NMDA-treated, and NMDA-treated with a single dose of omega-agatoxin-Aa2a) for SY1A (*P* < 0.001), SYT1 (*P* < 0.001), and SYN (*P* < 0.0001).

### Mossy fiber circuit LTP

**FIGURE 10:**
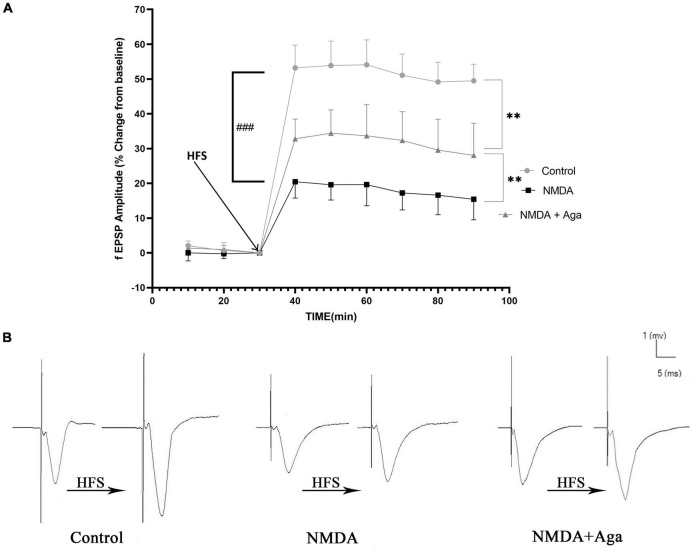
Mossy Fiber circuit LTP. **(A)** Long-term potentiation (LTP) curves of the field excitatory postsynaptic potential (fEPSP) amplitude in the hippocampal CA_3_ for the all groups (*n* = 6). The data are shown as means ± SEM of 6 rats per group. (*P < 0.05,^**^P < 0.01, ^***^P < 0.001, and ^****^P < 0.0001), and (^###^*P* < 0.001). **(B)** Sample traces of typical recorded fEPSPs in the hippocampal CA_3_ neurons before and after high-frequency stimulation (HFS) induction for the long-term potentiation (LTP) in experimental groups.

According to the Figure 10A, the injection of NMDA significantly decreased the field excitatory postsynaptic potentials (fEPSP) amplitude in the NMDA-treated group after LTP induction in the CA_3_ area of the hippocampus when compared to the control group (*P* < 0.001). Administration of a single dose of omega-agatoxin-Aa2a after the injection of NMDA in the NMDA-treated + Aga group remarkably increased the fEPSP amplitude after LTP induction with respect to the NMDA-treated group (*P* < 0.01). The fEPSP amplitude in the NMDA-treated + Aga group represented a significant difference compared to the control group (*P* < 0.01).

Figure 10B, demonstrates the traces of the recorded fEPSPs in the hippocampal CA_3_ neurons, before and after LTP induction using the high-frequency stimulation (HFS) technique in all the compared groups.

### Localize SNAP-25 in CA_3_ sub-region of the hippocampus

**FIGURE 11:**
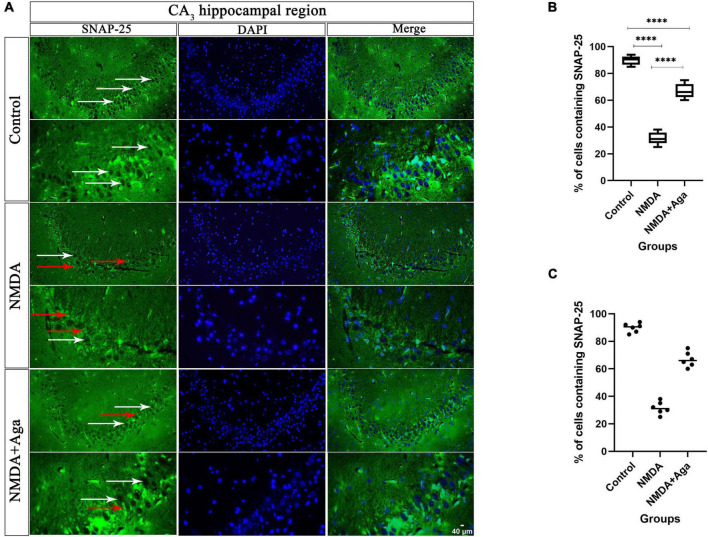
Immunofluorescence staining with antibody against SNAP-25. **(A)** The localize SNAP-25 are presented in the CA_3_ area of rat hippocampus. DAPI was applied to counterstain the nuclei. The healthy (white arrow) and dead (red arrow) pyramidal neurons are indicate in the left column. **(B)** The localize SNAP-25 are Shown in the CA_3_ area of rat hippocampus for the experimental groups (In percent). The data are shown as means ± SEM of 3 rats per group. (*P < 0.05, ^**^P < 0.01, ^***^P < 0.001, and ^****^P < 0.0001). **(C)** Scatter plot of part label **(B)**.

Our data showed that the single injection of NMDA significantly decreased the expression of SNAP-25 protein in the CA_3_ area of the hippocampus compared to the control group (*P* < 0.0001). The single administration of omega-agatoxin-Aa2a after injection of NMDA distinctly enhanced the expression of SNAP-25 in the CA_3_ area of rat hippocampus with respect to the NMDA group (*P* < 0.01). The NMDA + Aga group showed a significant differences in the CA_3_ sub-region of the rat hippocampus compared to the control group (*P* < 0.01; Figures 11A–C.

### Cresyl violet staining

**FIGURE 12:**
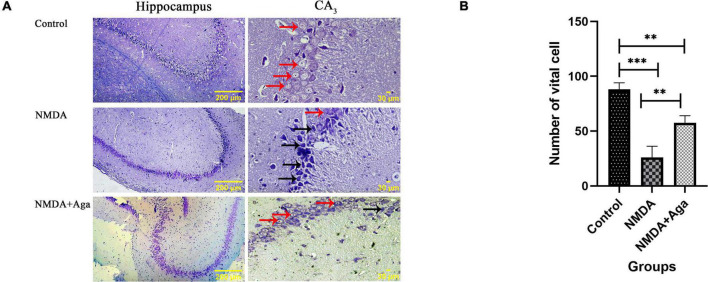
The cresyl violet staining of rat hippocampus. **(A)** The graph of healthy (Narrow red arrow) and dead (Narrow black arrow) pyramidal neurons in the CA3 sub-region of the rat hippocampus. **(B)** The number of healthy cells are shown in the CA3 sub-region of the rat hippocampus. The data are shown as means ± SEM of 3 rats per group. (*P < 0.05, ^**^P < 0.01, ^***^P < 0.001, and ^****^P < 0.0001).

Our data showed that the single injection of NMDA significantly decreased the expression of living pyramidal neurons in the CA_3_ sub-region of the hippocampus compared to the control group (*P* < 0.001). The single administration of omega-agatoxin-Aa2a after injection of NMDA, distinctly enhanced the living pyramidal neurons in the CA_3_ sub-region of the hippocampus with respect to the NMDA-treated group (*P* < 0.01). The NMDA-treated + ligand group showed a significant difference in the CA_3_ sub-region of the hippocampus compared to the control group (*P* < 0.01; Figures 12A, B).

## Discussion

In the current study, the omega-agatoxin-Aa2a was purified and identified from *Agelena labyrinthica* crude venom. This species is a sister group of *Agelenopsis aperta* (Wheeler et al., 2017). The specimens were collected from Iran. After the identification of specimens using the taxonomic key, particularly the epigyne, the venom glands were separated and crude venom was extracted from them. Amount and protein concentration of crude venom were in agreement with a previously published by Beneli and Yigit (2008). The crude lyophilized crude venom was a white powder with a normal appearance. A suitable buffer under low-pressure condition was used to purify the omega-agatoxin-Aa2a from the crude venom. Therefore, the native structure of the ligand was protected from degradation. At each step of the purification process, gel-electrophoresis was performed, to confirm the purification process. Based on the collected data from the LD_50_ section, the toxicity of this species crude venom was low and the omega-agatoxin-Aa2a protein LD_50_ was far less than the crude venom. This decrease in LD_50_ could be caused due to the separation of the crude venom. Crude venom act as a single compartment. Although venoms have multiple parts, they can operate perfectly when these parts act together. Therefore, a decrease in the potency of each part of a venom following separation of crude venom seems logical. On the other hand, the enzymatic part of a crude venom has a high molecular weight whereas; the omega-agatoxin-Aa2a protein has a low molecular weight (Keimasi et al., 2022). Accordingly, the omega-agatoxin-Aa2a protein is safe, particularly in the microliter concentration, which has been used in the current study.

The omega-agatoxin-Aa2a is a state-dependent small protein that has interacts with N-type VGCCs, according to the Sousa et al. (2013) study. This ligand can potentially block the N-type VGCCs. The omega-agatoxin-Aa2a for the first time has been purified from *Agelenopsis aperta* species for the first time (Herzig et al., 2010; Consortium, 2019). Protein measurement of omega-agatoxin-Aa2a was performed for dosage determination.

Ion channels have a crucial role in signal transduction and convert electrical neurotransmission to chemical neurotransmission (Simms and Zamponi, 2014). The last location of the neurotransmission in the neurons is presynaptic axon terminal. Calcium channels in this area are voltage-gated-type (Xu and Tang, 2018). These channels include N, and P/Q VGCCs (Zamponi and Snutch, 1998). A nerve impulse opens the alpha1-segment of VGCCs, and calcium ions enter into the presynaptic terminal and trigger the release of neurotransmitters (Nimmrich and Gross, 2012). As said before, glutamate is the main hippocampus neurotransmitter. Therefore, the release of this excitatory neurotransmitter can be modulated by an appropriate ligand. The omega-agatoxin-Aa2a as a ligand can bind to the alpha1-segment of N-type VGCCs (Sousa et al., 2013). This is important, as in neurodegeneration diseases like AD the structure of the ion channels can be transformed by amyloid beta which leads to the malfunction of channels and finally ends to an overload of calcium in presynaptic neurons and releasing high amounts of glutamate in the synaptic cleft (Koutsilieri and Riederer, 2007; Szydlowska and Tymianski, 2010; Zhang et al., 2015). In such condition, excitotoxicity can be induced and its consequences lead to neuronal elimination through apoptosis and necrosis pathways (Belousov, 2012; Verma et al., 2022). It is obvious that the omega-agatoxin-Aa2a is efficient as a ligand in excitotoxicity condition, because of its binding site.

As soon as an action potential reaches to the presynaptic area, the calcium ions enter the neurons (Mochida, 2019). SNAP-25, SY1A, SYT1, and SYN are synaptic markers (Li and Kavalali, 2017). SNAP-25 has a fundamental role in synaptic function and transmission of a signal from the presynaptic neuron to postsynaptic neuron (Zhang et al., 2014; Li and Kavalali, 2017). The SY1A has a straight connection *via* SNAP-25 (Ullrich et al., 2015). Therefore, the SY1A has a fundamental role in the docking vesicles into the presynaptic membrane and trigger of neurotransmitter release (Ullrich et al., 2015; Kim and Oh, 2016; Li and Kavalali, 2017). The SYT1 is a calcium-sensitive sensor, which has a crucial role in the fast vesicle exocytosis of neurotransmitters from neurons (Zhang et al., 2014; Hussain et al., 2017). The SYN is a major synaptic vesicle protein, which is localized in the presynaptic neurons. SYN has an important role in vesicular docking and neurotransmitter release (Zhang et al., 2014; Ji et al., 2017). Our data indicated that administration of NMDA in rat hippocampus could induces excitotoxicity due to over-stimulation of NMDARs, which in turn reduces SNAP-25 expression protein level. Also, this administration reduced SY1A, SYT1, and SYN mRNAs expression (Yu et al., 2006; Li et al., 2012; You et al., 2018; Keimasi et al., 2022). This reduction of SNAP-25, SY1A, SYT1, and SYN leads to cognitive impairment and memory dysfunction, which can be reversed by an increase in SNAP-25, SY1A, SYT1, and SYN following the blockage of presynaptic N-type VGCCs with omega-agatoxin-Aa2a. The function of SNAP-25, SY1A, SYT1, and SYN relies on presynaptic VGCCs. Normal rates of SNAP-25, SY1A, SYT1, and SYN ends in neurotransmitter release in the synaptic cleft (Valtorta et al., 2004; Tampellini et al., 2010; Zhang et al., 2014; Biswal et al., 2017). The release of glutamate in hippocampus neurons can induce LTP and neuron plasticity (Bin Ibrahim et al., 2022). Therefore, the presence of SNAP-25, SY1A, SYT1, and SYN is essential for memory and learning. The reduction or loss of SNAP-25, SY1A, SYT1, and SYN expressions causes memory impairment and cognitive deficit in AD. Thus, the function of SNAP-25, SY1A, SYT1, and SYN is critical for synaptic performance (Li and Kavalali, 2017). However, the blockage of N-type VGCCs by omega-agatoxin-Aa2a as a ligand was not able to restore the SNAP-25, SY1A, SYT1, and SYN expressions to the normal levels.

Based on the collected data from cresyl violet staining, the NMDA injection into the CA_3_ sub-region of the hippocampus eliminated the pyramidal neurons in this area through neurodegeneration, induced by the excitotoxicity process. The administration of omega-agatoxin-Aa2a after NMDA injection had an ameliorative effect on neurodegeneration *via* blocking the N-type VGCCs and controlling the excitotoxicity process through the regulation of neurotransmitters release (Sousa et al., 2013; Mochida, 2019). Glutamate has an undeniable role in memory and learning performance. However, the hyper-activity of this neurotransmitter can induce neurodegeneration through the hyper-stimulation of the NMDA receptors (Dong et al., 2009; Crews and Masliah, 2010). Therefore, agonists of NMDAR like as NMDA, alpha-amino-3-hydroxy-5-methyl-4-isoxazolepropionic acid (AMPA) and kainic acid can induce excitotoxicity, according to multiple studies (Jarrard, 2002; Zhang et al., 2014, 2015; Hosseini-Sharifabad et al., 2021; Naseri et al., 2022). In addition, NMDA has been used for the induction of cognitive impairment, learning, and memory defects. Our data demonstrated that intra-hippocampal injection of NMDA eliminated the memory and learning performance, which is in line with the previous study (Jarrard, 2002).

The collected results from the Morris water maze and Passive Avoidance tests showed defects in cognitive memory, and learning and memory performance. The effect of NMDA treatment appeared in the rat’s behavior. The NMDA-treated rats had a critical problem to locate the hidden platform and failed to complete this task. Plus, these rats were not able to discriminate the target zone from other zones. Therefore, this group had a serious problem with cognitive memory and learning function (Ebrahimpour et al., 2018). Despite this, the NMDA-treated group who had received a single dose of omega-agatoxin-Aa2a was able to finish the task. Therefore, the effect of this ligand on the N-type VGCCs was related to cognitive memory and learning performance. Memorizing function relies on LTP process, which has various types in the hippocampus. The main types of LTP include postsynaptic, presynaptic, and both pre and postsynaptic. LTP process induces the memory formation. The pyramidal neurons in the CA_3_ sub-region of the hippocampus have mostly presynaptic LTP. These particular neurons are highly connected together, and are able to excite each other. Another feature of these pyramidal neurons is their large presynaptic terminals and frequency of their neurotransmitter release sites. Therefore, it seem logical for LTP in Mossy Fiber be presynaptic and dependent to calcium influx into the neurons. In other words, the LTP in Mossy Fiber is non-associative (Alkadhi, 2021). The mentioned features in the CA_3_ sub-region of the hippocampus make this area a perfect place for excitotoxicity induction (because of the self-excision ability) and evaluation of the effect of omega-agatoxin-Aa2a as an N-type VGCCs ligand.

As a conclusion, the induced excitotoxicity by NMDA resulted in pyramidal neuron death in the CA_3_ sub-region of the hippocampus, reduction of fEPSP after LTP induction, decrease in the rate of SNAP-25 protein, and downregulation of SY1A, SYT1, and SYN mRNAs, as well as cognitive and learning memory performance elimination. However, a single injection of omega-agatoxin-Aa2a in the NMDA-treated rats led to the prevention of consequences of excitotoxicity, through the blockage of N-type VGCCs. In addition, a single injection of omega-agatoxin-Aa2a could reverse the cognitive memory and learning impairment, upregulate SNAP-25 protein, SY1A, SYT1, and SYN mRNA levels in the CA_3_ sub-region of the hippocampus, enhance the fEPSP after LTP induction, and prevent pyramidal neuron death. This study can be considered as a starting point for future study on evaluation of the effect of omega-agatoxin-Aa2a on the cognitive abilities such as; pain sensitivity, sensorimotor, and locomotor ability. In addition, evaluation of the effects of bioactive small proteins on the N-type and P/Q-types VGCCs as a co-treatment can be suggested for the next research.

## Data availability statement

The original contributions presented in the study are included in the article/supplementary material further inquiries can be directed to the corresponding authors. Requests to access these datasets should be directed to mofid@pharm.mui.ac.ir, moradmand.arachnids@gmail.com, and keimasimohammed@gmail.com.

## Ethics statement

The animal study was reviewed and approved by the animal Ethics Committee of the University of Isfahan.

## Author contributions

MK conceived the original idea. MK, MM, and MRM planned the experiments. MK, KS, MJK, MA, NE, and FE performed the experiments, data collection, analysis, and interpretation. MK and MA wrote the manuscript. MM, MRM, and MA have collaborated in presenting the research idea. MK, MM, and MRM supervised the project. All authors revised the manuscript and approved the final version of manuscript.
