# Corrigendum: Alleviation of cognitive deficits in a rat model of glutamate-induced excitotoxicity, using an N-type voltage-gated calcium channel ligand, extracted from *Agelena labyrinthica* crude venom

**DOI:** 10.3389/fnmol.2023.1180964

**Published:** 2023-03-16

**Authors:** Mohammad Keimasi, Kowsar Salehifard, Mohammadjavad Keimasi, Mohammadreza Amirsadri, Noushin Mirshah Jafar Esfahani, Majid Moradmand, Fariba Esmaeili, Mohammad Reza Mofid

**Affiliations:** ^1^Department of Plant and Animal Biology, Faculty of Biological Sciences and Technology, University of Isfahan, Isfahan, Iran; ^2^Department of Physiology, School of Medicine, Isfahan University of Medical Sciences, Isfahan, Iran; ^3^Department of Clinical Pharmacy and Pharmacy Practice, School of Pharmacy and Pharmaceutical Sciences, Isfahan University of Medical Sciences, Isfahan, Iran; ^4^Department of Clinical Biochemistry, School of Pharmacy and Pharmaceutical Sciences, Isfahan University of Medical Sciences, Isfahan, Iran

**Keywords:** cognitive dysfunction, spider, calcium channel Cav2.2 (N-type), learning and memory, venom

In the published article, there was an error in the “Figure 1B” as published. This picture was misplaced during the submission by one of the authors. The corrected “Figure 1” appears below.

**Figure 1:**
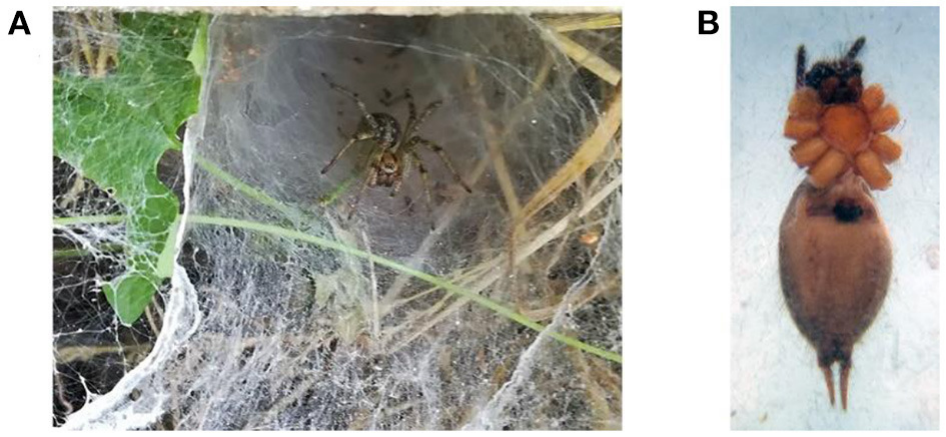
*Agelena labyrinthica* species. **(A)** The *Agelena labyrinthica* spider on the net. **(B)** The epigyne of female specimens.

The authors apologize for this error and state that this does not change the scientific conclusions of the article in any way. The original article has been updated.

